# Anti-Neuroinflammatory and Anti-Inflammatory Activities of Phenylheptatriyne Isolated from the Flowers of *Coreopsis lanceolata* L. via NF-κB Inhibition and HO-1 Expression in BV2 and RAW264.7 Cells

**DOI:** 10.3390/ijms22147482

**Published:** 2021-07-13

**Authors:** Hwan Lee, Zhiming Liu, Chi-Su Yoon, Linsha Dong, Wonmin Ko, Eun-Rhan Woo, Dong-Sung Lee

**Affiliations:** 1 College of Pharmacy, Chosun University, Dong-gu, Gwangju 61452, Korea; ghksdldi123@hanmail.net (H.L.); lzmqust@126.com (Z.L.); donglinsha011@163.com (L.D.); rabis815@naver.com (W.K.); wooer@chosun.ac.kr (E.-R.W.); 2 College of Pharmacy, Wonkwang University, Iksan 54538, Korea; ycs1991@naver.com

**Keywords:** *Coreopsis lanceolata* L., flower, mouse macrophage RAW 264.7, mouse microglia BV2, anti-inflammation

## Abstract

Aging is associated with immune disregulation and oxidative stress which lead to inflammation and neurodegenerative diseases. We have tried to identify the anti-neuroinflammatory and anti-inflammatory components of *Coreopsis lanceolata* L. The dried flowers of *C. lanceolata* were extracted with 70% EtOH, and the obtained extract was divided into CH_2_Cl_2_, EtOAc, *n*-BuOH, and H_2_O fractions. The CH_2_Cl_2_ fraction was separated using silica gel and C-18 column chromatography to yield phenylheptatriyne (**1**), 2′-hydroxy-3,4,4′-trimethoxychalcone (**2**), and 4′,7-dimethoxyflavanone (**3**). Additionally, the EtOAc fraction was subjected to silica gel, C-18, and Sephadex LH-20 column chromatography to yield 8-methoxybutin (**4**) and leptosidin (**5**). All the compounds isolated from *C. lanceolata* inhibited the production of nitric oxide (NO) in LPS-induced BV2 and RAW264.7 cells. In addition, phenylheptatriyne and 4′,7-dimethoxyflavanone reduced the secretion of inflammatory cytokines, tumor necrosis factor alpha (TNF-α), and interleukin (IL)-6. Among them, phenylheptatriyne was significantly downregulated in the expression of inducible NO synthase (iNOS) and cyclooxygenase-2 (COX-2). Subsequently, phenylheptatriyne also effectively inhibited nuclear factor-kappa B (NF-κB) activation in LPS-stimulated BV2 and RAW264.7 cells. Based on these results, the anti-neuroinflammatory effect of phenylheptatriyne isolated from *C. lanceolata* was confirmed, which may exert a therapeutic effect in treatment of neuroinflammation-related diseases.

## 1. Introduction

As the average life span of humans is increasing, aging societies are rapidly developing in many countries, and the attitude towards prevention of aging is actively changing worldwide. Aging has been linked to immune disregulation, oxidative stress, and free radical generation [1]. In addition, it is accompanied by a decrease in organ function and loss of regenerative ability [2]. Among these dysfunctions, oxidative stress contributes to an aging-related chronic inflammatory process, called “inflammatory aging” [3]. Inflammation is a phenomenon in which pro-inflammatory mediators, such as cytokines and chemokines, are synthesized and released [4]. Chronic inflammation is a condition in which inflammatory cytokines and chemokines are increased in response to physiological and environmental factors, allowing the immune system to continue functioning at low levels [5]. The causes of chronic inflammation include genetic susceptibility, visceral obesity, chronic infection, and cellular aging [6]. Oxidative stress contributes to the development of age-related diseases, causes imbalance in the body, and directly causes oxidative damage to cells, resulting in an inflammatory response in which local tissues resist the invasion of chemical or biological factors [7,8,9,10]. During the inflammatory reaction to oxidative stress, unnecessary reactive oxygen species (ROS), such as singlet oxygen (^1^O_2_), superoxide (O_2_^−^), hydroxyl radical (OH), and hydroxyl peroxide (H_2_O_2_), cause disorders of nucleic acids, proteins, and lipids, and block normal functions [11,12]. Furthermore, in this process, inflammation occurs due to DNA damage, cell death, and aging-related diseases, such as degenerative brain disease and cardiovascular disease, thereby causing skin aging [13].

*Coreopsis lanceolata* is a plant of the Compositae family. Compositae plants have long been used to treat cold, headache, and high blood pressure. Numerous natural compounds have been isolated from Compositae family plants, and many flavonoid-type compounds have been reported [14,15,16]. *C. lanceolata* also has antioxidant and anticancer effects, and contains various flavonoids known to lower blood sugar [16]. However, although some research on *C. lanceolata* is ongoing, research investigating its anti- neuroinflammatory and anti-inflammatory properties is sparse. In particular, studies on the isolation of the active ingredient of *C. lanceolata* and the damage caused by inflammation are still insufficient. Therefore, in this study, the active ingredient was isolated from *C. lanceolata* and its anti-inflammatory effects were investigated on BV2 microglia and RAW264.7 macrophages by using various in vitro models.

## 2. Results

### 2.1. Isolation and Identification of Flavonoids from C. lanceolata Flowers

**Figure 1:**
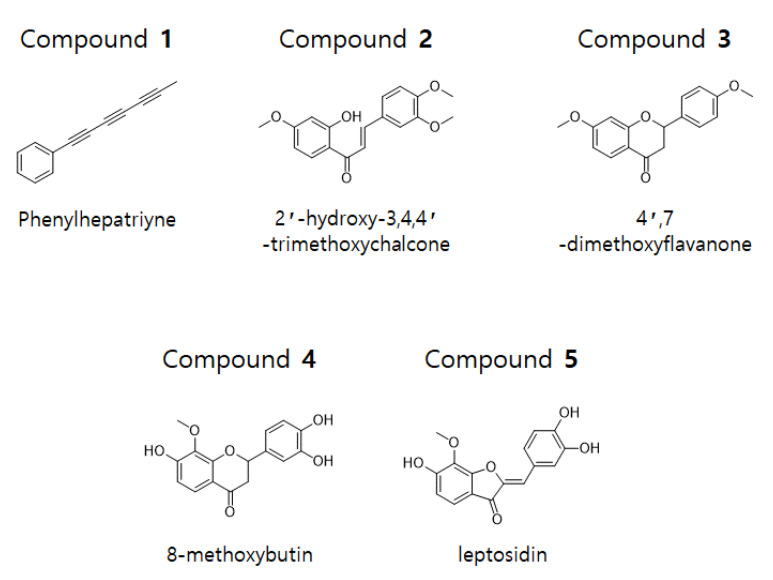
Chemical structure of compounds **1**–**5** isolated from *C. lanceolata* flowers.

The dried flowers of *C. lanceolata* were extracted with 70% EtOH and fractionated with CH_2_Cl_2_, EtOAc, *n*-BuOH, and H_2_O. The CH_2_Cl_2_ and EtOAc fractions were subjected to silica gel, C-18, and Sephadex LH-20 column chromatography to obtain five compounds. The isolated compounds were analyzed by 1D-NMR (^1^H, ^13^C, DEPT) and 2D-NMR (COSY, HSQC, HMBC) spectroscopy. Phenylheptatriyne (1), 2′-hydroxy-3,4,4′-trimethoxychalcone (2), and 4′,7-dimethoxyflavanone (3) were structurally identified in the CH_2_Cl_2_ fraction and 8-methoxybutine (4) and leptosidine (5) were structurally identified in the EtOAc fraction (Figure 1).

Compound **1** was obtained as a yellow gum. Analysis of the ^1^H- and ^13^C-NMR (CDCl_3_) data revealed the molecular formula to be C_13_H_8_. The ^1^H-NMR spectrum of compound **1** revealed five aromatic protons (δ 7.51–7.49 and 7.39–7.29) and a methyl proton (δ = 2.0). The ^13^C-NMR and DEPT data revealed five olefinic carbons (δ 128.5, 129.5, and 133.0), an aromatic quaternary carbon (δ 121.2), six sp carbons (δ 59.0, 65.0, 67.5, 74.7, 75.3, and 78.3), and methyl carbon (δ 4.7). From the analysis of ^1^H- and ^13^C-NMR data, the structure of this compound was conjectured to have a methyltriacetylene group-attached phenyl group. From the comparison with ^1^H- and ^13^C-NMR data in the literature, the structure of compound **1** was determined to be hepta-1,3,5-triynylbenzene (phenylheptatriyne) [17].

Compound **2** was obtained as a yellow gum. Analysis of the ^1^H- and ^13^C-NMR (CDCl_3_) data revealed the molecular formula to be C_18_H_18_O_5_. The ^1^H-NMR data of compound **2** revealed a hydroxyl group (δ 13.54), two *trans* olefinic protons (δ 7.44, 7.86, and 15.6 Hz), six olefinic protons (δ 6.49, 6.50, 6.91, 7.17, 7.26, and 7.84), and three methoxy protons (δ 3.87, 3.94, and 3.97). The ^13^C-NMR data showed the presence of 18 signals, including a carbonyl carbon (δ 191.8), four oxygenated aromatic carbons (δ 149.3, 151.6, 166.1, and 166.6), two α,β-unsaturated carbons (δ 144.6, 131.1), and three methoxy carbons (δ 55.6, 56.00, and 56.02). From the analysis of ^1^H- and ^13^C-NMR data, the structure of this compound was conjectured to have a chalcone skeleton. Further analysis of the HMBC data confirmed the position of the methoxy and hydroxy groups. From the comparison with ^1^H- and ^13^C-NMR data from previous literature, the structure of compound **2** was determined to be (*E*)-3-(3,4-dimethoxyphenyl)-1-(2-hydroxy-4-methoxyphenyl)prop-2-en-1-one (2′-hydroxy-3,4,4′-trimethoxychalcone) [18].

Compound **3** was obtained as a yellow gum. Analysis of the ^1^H- and ^13^C-NMR (CDCl_3_) data revealed the molecular formula to be C_17_H_16_O_4_. The ^1^H-NMR data of compound **3** showed seven olefinic protons [δ 6.48, 6.62, 6.96 (2H), 7.41 (2H), and 7.87], an oxygenated methine proton (δ 5.42), two methoxy protons (δ 3.84, 3.83), and a methylene proton (δ 3.06, 2.80). The ^13^C-NMR data showed the presence of 17 signals, including a carbonyl carbon (δ 190.9), three oxygenated aromatic carbons (δ 159.9, 163.6, and 166.1), an oxygenated methine carbon (δ 79.8), two methoxy carbons (δ 55.4 and 55.6), and a methylene carbon (δ 44.1). From the analysis of the ^1^H- and ^13^C-NMR data, the structure of this compound was conjectured to have a flavanone skeleton. Based on comparison with ^1^H- and ^13^C-NMR data in the literature, the structure of compound **3** was determined to be 7-methoxy-2-(4-methoxyphenyl)chromen-4-one (4′,7-dimethoxyflavanone) [19].

Compound **4** was obtained as a red gum. Analysis of the ^1^H- and ^13^C-NMR (DMSO-*d_6_*) data revealed the molecular formula to be C_16_H_14_O_6_. The ^1^H- and ^13^C-NMR data of compound **4** suggested that, except for a missing methoxy group and three added hydroxyl groups, its structure was similar to that of compound **3**. From the analysis of HMBC data, unlike compound **3**, this compound was found to have two hydroxy groups attached at positions 7′ and 3′, while the methoxy group at position 4′ was substituted with a hydroxy group. From the comparison with ^1^H- and ^13^C-NMR data from previous literature, the structure of compound **4** was determined to be 2-(3,4-dihydroxyphenyl)-7-hydroxy-8-methoxy-2,3-dihydrochromen-4-one (8-methoxybutin) [20].

Compound **5** was obtained as a red gum. Analysis of the ^1^H- and ^13^C-NMR (DMSO-*d_6_*) data revealed the molecular formula to be C_16_H_12_O_6_. The ^1^H-NMR data of compound **5** showed three hydroxy groups (δ 9.35, 9.73, and 10.84), six olefinic protons (δ 6.68, 6.78, 6.86, 7.26, 7.34, and 7.45), and a methoxy proton (δ 4.03). The ^13^C-NMR data showed the presence of 16 signals, including a carbonyl carbon (δ 181.9), five oxygenated olefinic carbons (δ 146.05, 146.14, 148.8, 158.3, and 158.6), and a methoxy carbon (δ 61.3). From the analysis of ^1^H- and ^13^C-NMR data, the structure of this compound was conjectured to have a skeleton of aurone. Further HMBC analysis confirmed the presence of three hydroxy and methoxy groups. Based on comparison with ^1^H- and ^13^C-NMR data from previous literature, the structure of compound **5** was determined to be (2*Z*)-2-[(3,4-dihydroxyphenyl)methylidene]-6-hydroxy-7-methoxy-1-benzofuran-3-one (leptosidin) [20].

### 2.2. Inhibitory Effect of the Five Natural Compounds Isolated from C. lanceolata Flowers on Nitrite and PGE_2_ Production

Macrophages and microglia are activated in the body in the presence of harmful stimuli and play an important role in maintaining homeostasis in the body. However, when abnormally activated by external stimuli, pro-inflammatory mediators, ROS, and inflammatory cytokines are secreted [21]. The secretion of inflammatory mediators can lead to cell and brain damage, leading to degenerative brain diseases, such as Alzheimer’s and Parkinson’s disease [22,23]. When macrophages and microglia are activated by external stimuli, pro-inflammatory substances such as nitric oxide (NO) and prostaglandin E2 (PGE_2_) are secreted. Suppressing the secretion of active inflammatory substances can prevent cell damage. We, therefore, evaluated the neuroinflammation inhibitory effect of the compounds on BV2 microglia and RAW264.7 macrophage induced by LPS.

**Figure 2:**
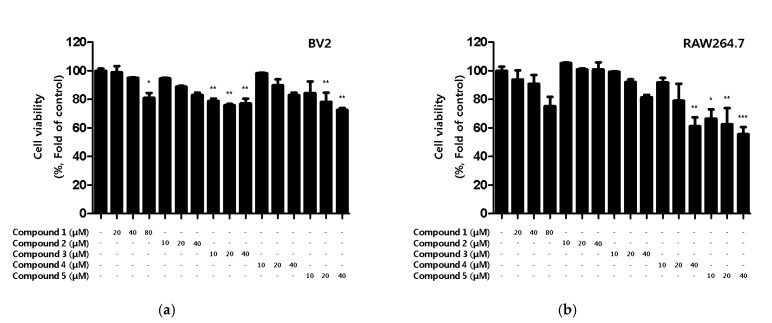
Cytotoxicity of the five natural compounds isolated from *C. lanceolata* flowers in BV2 microglia (**a**) and RAW264.7 macrophages (**b**). Cytotoxicity was evaluated in cells treated for 48 h with 10 to 80 μM of compounds **1**–**5**. Data are presented as the mean ± SD values of 3 independent experiments. * *p* < 0.05, ** *p* < 0.01, *** *p* < 0.001 vs. control.

First, the toxicity of the compounds was assessed. Although toxicity was not observed in BV2 microglia for any of the compounds, toxicity was observed for compound **4** and **5** in RAW264.7 macrophages (Figure 2).

**Figure 3:**
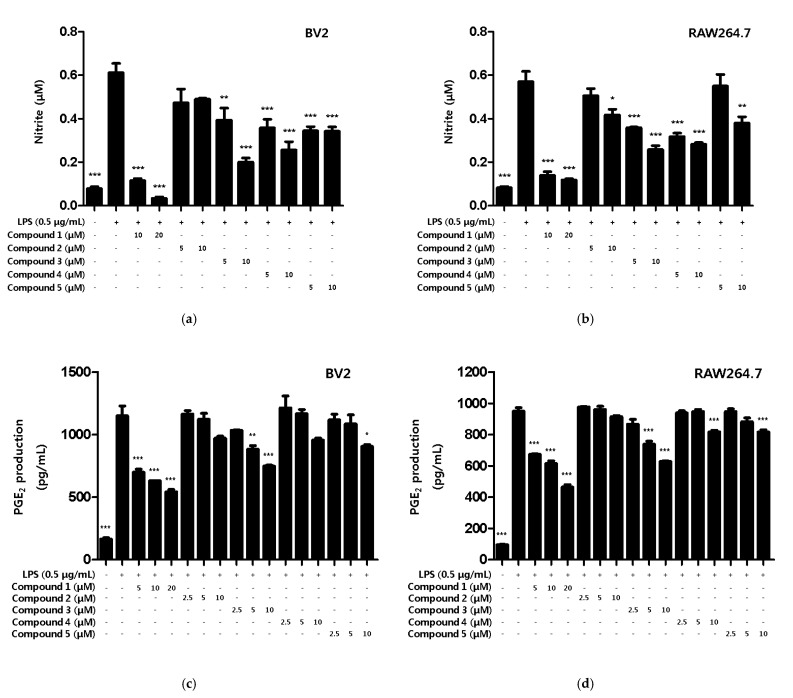
The inhibitory effect of compounds on the nitrite and PGE_2_ production in BV2 microglia and RAW264.7 macrophages. The inhibitory effect of nitrite production in BV2 (**a**) and RAW264.7 (**b**) cells was induced for 18 h by pre-treatment with 5–20 μM of compounds **1**–**5** and then treatment with LPS. The inhibitory effect of PGE_2_ production in BV2 (**c**) and RAW264.7 (**d**) cells was induced for 18 h by pre-treatment with 2.5–20 μM of compounds **1**–**5** and then treatment with LPS. Data is expressed as the mean ± SD value of 3 independent experiments. * *p* < 0.05, ** *p* < 0.01, *** *p* < 0.001 vs. LPS.

Based on the results of the toxicity evaluation, the treatment concentration of the compound was set to a maximum of 20 μM for compound **1** or 10 μM for compounds **2**, **3**, **4**, and **5** to confirm the inhibitory effect of nitrite production. In LPS-induced BV2 microglia and RAW264.7, all of the compounds, except compound **2**, showed inhibitory effects on nitrite production, with compound **1** showing the greatest inhibitory effect (Figure 3a,b). In addition, the inhibitory effect of the compounds on PGE_2_ production was evaluated. Among the compounds, only compound **1** inhibited the production of PGE_2_ in BV2 microglia and RAW264.7 macrophages (Figure 3c,d).

### 2.3. Inhibitory Effects of the Five Natural Compounds Isolated from C. lanceolate Flowers on Cytokine Production

**Figure 4:**
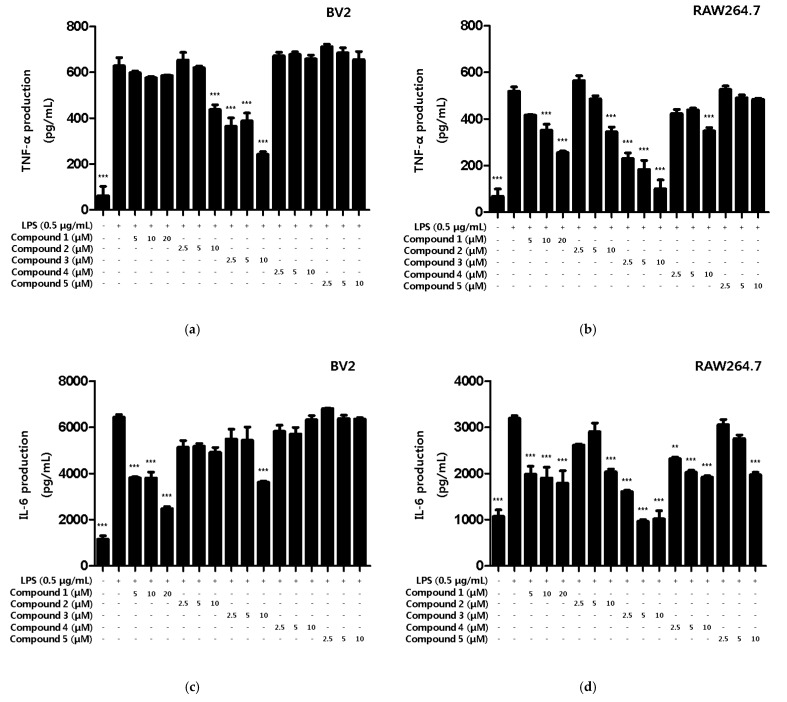
Inhibitory effects of inflammatory cytokines TNF-α and IL-6 on compounds in BV2 microglia and RAW264.7 macrophages. The inhibitory effect of TNF-α production in BV2 (**a**) and RAW264.7 (**b**) cells was induced for 18 h by pre-treatment with 2.5–20 μM of compounds **1**–**5**, followed by treatment with LPS. The inhibitory effect of IL-6 production in BV2 (**c**) and RAW264.7 (**d**) cells was induced for 18 h by pre-treatment with 2.5–20 μM of compounds **1**–**5**, followed by treatment with LPS. Data is expressed as the mean ± SD value of 3 independent experiments. ** *p* < 0.01, *** *p* < 0.001 vs. LPS.

Various intracellular inflammatory cytokines such as TNF-α, IL-1β, IL-6 and prostaglandins function as mediators of several inflammatory modulators [24]. However, when cells are activated by external stimuli, cytokines are excessively secreted to modulate the upregulation of inflammation [25]. In the present study, we observed the inhibitory effect of the compounds on inflammatory cytokine production in LPS-induced BV2 microglia and RAW264.7 macrophages. First, the inhibitory effects of compounds on the TNF-α production were measured. All compounds, except compound **5**, were found to inhibit TNF-α production in RAW264.7, and compounds **2** and **3** showed inhibitory effects on the TNF-α production in BV2 microglia (Figure 4a,b). Next, the inhibitory effect of compounds on IL-6 production was measured. As a result, all compounds inhibited IL-6 production in RAW264.7, and compound **1** and **3** inhibited the production of BV2 microglia (Figure 4c,d). Therefore, we wanted to conduct additional mechanistic studies of compounds **1** and **3**, however, since the amount of compound **3** obtained in this study was only 0.8 mg, further experiments were conducted using only compound **1**.

### 2.4. Inhibitory Effect of Phenylheptatriyne on iNOS and COX-2 Expression

**Figure 5:**
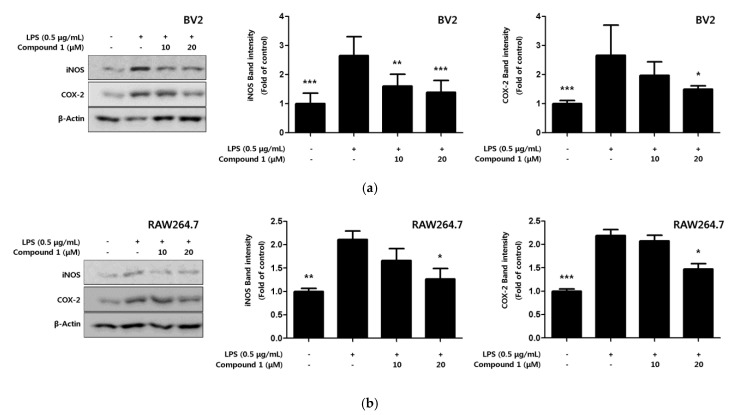
Effect of compound **1** on protein expression levels of nitric oxide synthase (iNOS) and cyclooxygenase-2 (COX-2) in LPS-induced BV2 microglia (**a**) and RAW264.7 macrophages (**b**). The inhibitory effect on the expression of the pro-inflammatory proteins iNOS and COX-2 was induced in cells for 18 h by LPS treatment after pretreatment with 10–20 μM of compound **1**. Representative blots of 3 independent experiments, obtained by Western blot analysis, are shown. Data is expressed as the mean ± SD value of 3 independent experiments. * *p* < 0.05, ** *p* < 0.01, *** *p* < 0.001 vs. LPS.

Inflammatory cytokines and NO are active inflammatory substances that are rapidly produced when macrophages are activated in the presence of LPS or pathogens [26]. The production of these inflammatory substances increased by the pro-inflammatory proteins inducible nitric oxide synthase (iNOS) and cyclooxygenase-2 (COX-2). In the present study, the expression levels of pro-inflammatory proteins iNOS and COX-2 in response to compound **1**, which exhibits the strongest inhibitory effect on inflammatory cytokines, were confirmed through Western blot analysis. Compound **1** inhibited the expression of iNOS and COX-2 in a concentration-dependent manner (Figure 5a,b).

### 2.5. Inhibitory Effect of Phenylheptatriyne on NF-κB Translocation

**Figure 6:**
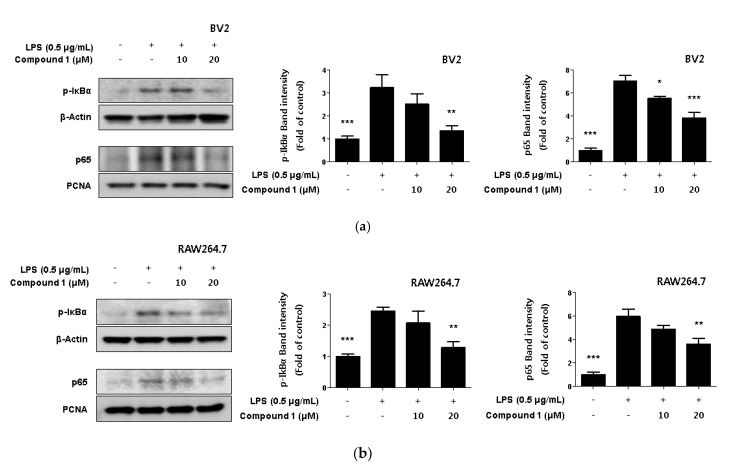
Inhibitory effect of compound **1** on Nuclear NF-κB translocation in LPS-induced BV2 microglia (**a**) and RAW264.7 macrophages (**b**). Cells were treated with 10–20 μM of compound **1** in the presence of 0.5 μg/mL LPS for 18 h. Western blot analysis was performed, and representative blots of 3 independent experiments are shown. Data is expressed as the mean ± SD value of 3 independent experiments. * *p* < 0.05, ** *p* < 0.01, *** *p* < 0.001 vs. LPS.

NF-κB is a transcription factor that regulates iNOS and COX-2 expression [27]. Normal NF-κB remains in its inactive form by forming complexes with regulatory proteins such as IκBα. However, when NF-κB is activated by LPS, IκBα is degraded by phosphorylation, and NF-κB is translocated to the nucleus [28]. There, it binds to the promoter of the inflammatory mediator gene and induces the expression of inflammatory mediators [29]. In Figure 5, we were found to inhibit the expression of pro-inflammatory proteins by compound **1**. Therefore, we were checked whether compound **1** regulated the NF-κB activation. As a result, it was confirmed that compound **1** inhibited p-IκBα phosphorylation in a concentration-dependent manner. Additionally, the translocation of p65 to the nucleus was inhibited in a concentration-dependent manner (Figure 6).

### 2.6. The Expression of HO-1 using by Phenylhpetatriyne and Inhibitory Effect of Phenylhpetatriyne on Nitrite Production through the regulation of HO-1 Activity

**Figure 7:**
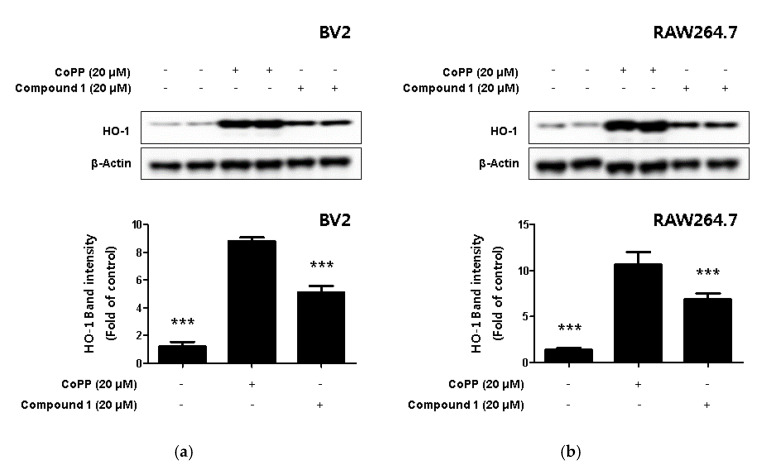
The effect of HO-1 expression in compound **1**-induced BV2 microglia (**a**) and RAW264.7 macrophages (**b**). CoPP was used as positive control. Cells were treated with 20 μM of CoPP or compound **1** and then incubated for 12 h. Western blot analysis was performed, and representative blots of 4 independent experiments are shown. Data is expressed as the mean ± SD value of 4 independent experiments. *** *p* < 0.001 vs. LPS.

Hemeoxygenase-1 (HO-1), a target of nuclear factor E2-related factor 2 (Nrf2), a powerful antioxidant signaling system in cells, is an enzyme that promotes free iron in carbon monoxide (CO), billiberdin, and heme [30]. HO-1 reduces the production of inflammatory cytokines and promotes the production of anti-inflammatory cytokines [31]. In addition, HO-1 is known to play an important role in protecting cells from inflammation and oxidative stress but is also known to regulate nitrite production in activated macrophages [32,33]. In the present study, we used Western blotting to investigate whether compound **1** increased the expression level of HO-1. In this process, the HO-1-inducer cobalt protoporphyrin (CoPP) was used as a positive control. The results revealed that compound **1** upregulated the expression of HO-1 (Figure 7a,b).

**Figure 8:**
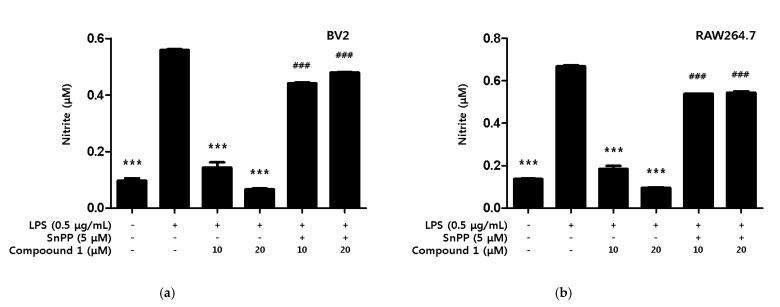
Inhibitory Effect of compound **1** on nitrite production through the regulation of HO-1 activity in BV2 microglia (**a**) and RAW264.7 macrophage (**b**). Cells were treated with 5 μM of SnPP and compound **1** and then exposed to LPS (0.5 μg/mL) for 18 h. Data are presented as mean ± SD values of 3 independent experiments. *** *p* < 0.001 vs. LPS. ^###^ *p* < 0.05 vs. LPS and Compound **1**.

Additionally, we investigated whether the inhibitory effect of compound **1** on nitrite production was dependent on HO-1. The investigation method used involved protoporphyrin IX (SnPP), a potent inhibitor of HO-1 activity, to determine whether HO-1 mediated the protective effect of compound **1** against the inflammatory response caused by LPS. The compound **1** treatment group showed a significant inhibitory effect on nitrite production in both LPS-induced BV2 microglia and RAW264.7, but the simultaneous treatment with SnPP showed no inhibitory effect on nitrite production (Figure 8).

## 3. Discussion

Inflammation is a biological response to infection and damage in certain tissues in the body, the main targets of which are immune cells. Inflammation is classified as acute or chronic according to the size of the affected area and the damage to the tissues of the body. When macrophages are stimulated by lipopolysaccharides (LPS), known as endotoxins, an inflammatory response occurs, and inflammatory cytokines such as interleukin (IL)-6 and IL-1β are released. In addition, inducible nitric oxide synthase (iNOS) and cyclooxygenase-2 (COX-2) expression trigger the inflammatory responses by producing various inflammatory mediators such as nitric oxide (NO) and prostaglandin E2 (PGE_2_) [34,35,36]. In recent years, functional natural compounds with secured stability have attracted the attention of the researchers, and research is being conducted to determine the utility of various natural products as functional natural compounds. Among these natural products, *Coreopsis lanceolata* is a plant of the family Compositae. Many natural compounds, most of them flavonoids, have been reported from the Compositae family. *C. lanceolata* has been reported to contain many flavonoids [14,15,16] and as having antioxidant and anti-cancer effects [16]. However, there are few studies on the anti-inflammatory effects of *C. lanceolata*, and in particular, the number of studies on the isolation of active compounds with anti-neuroinflammatory and anti-inflammatory effects are limited. In this study, we investigated the anti-neuroinflammatory and anti-inflammatory effects of five compounds isolated from the 70% EtOH extract of *C. lanceolata* flowers.

**Figure 9:**
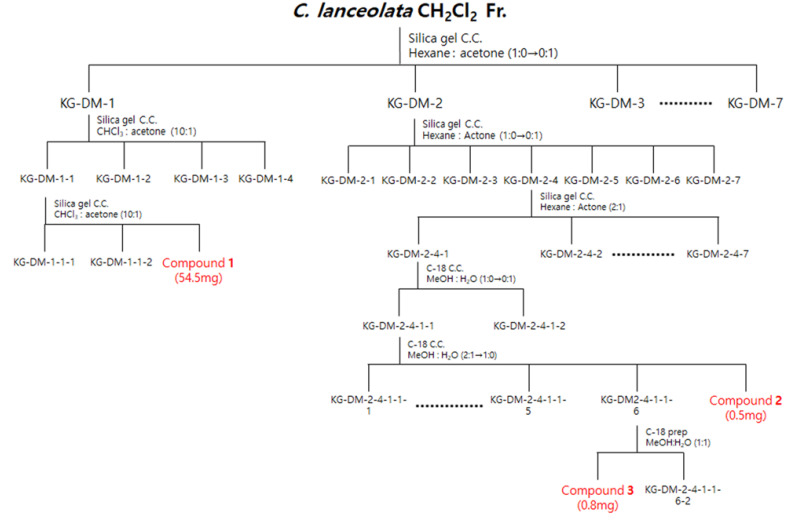
Scheme of the isolation of three compounds from the CH_2_Cl_2_ fraction of *C. lanceolata* L. 70% EtOH extract.

**Figure 10:**
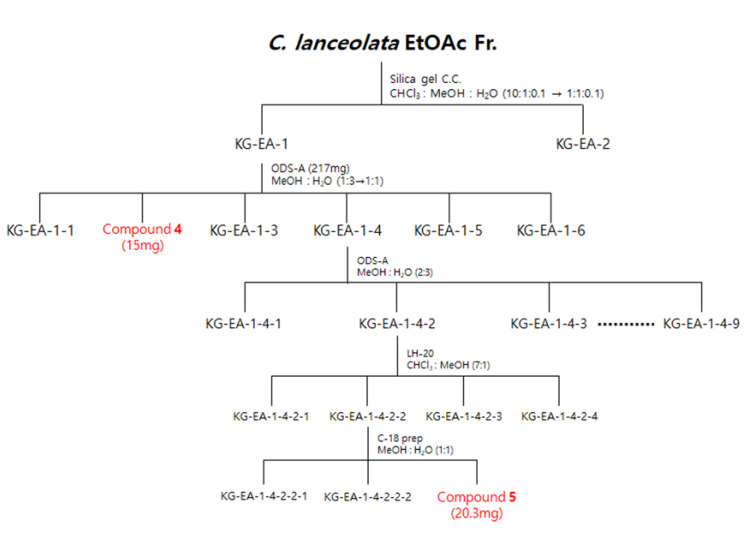
Scheme of the isolation of three compounds from the EtOAc fraction of *C. lanceolata* L. 70% EtOH extract.

The compounds isolated from *C. lanceolata* were identified to be phenylheptatriyne (**1**), 2′-hydroxy-3,4,4′-trimethoxychalcone (**2**), 4′,7-dimethoxyflavanone (**3**), 8-methoxybutine (**4**), and leptosidin (**5**) (Figure 1, Figure 9 and Figure 10). Phenylheptatriyne (**1**) has previously been obtained from *Bidens alba* var. Other reports have confirmed the antifungal, phototoxin, and antiviral effects of phenylheptatriyne isolated from *Bidens pilosa* L. [37,38,39]. However, in this work it was isolated for the first time from *C. lanceolata*, and thus far, there have been no reports of its anti-inflammatory activity. 2′-Hydroxy-3,4,4′-trimethoxychalcone (**2**) is a compound with a chalcone skeleton, and it has been reported to have many active effects, such as anti-cancer and anti-inflammatory effects [40,41]. 4′,7-Dimethoxyflavanone (**3**) is a compound with a flavanone skeleton, reported to have anticancer effects [42], however there are no reports on its anti-inflammatory activity. 8-Methoxybutine (**4**) and leptosidin (**5**) are known to have whitening and antioxidant effects and have been reported to be isolated from *C. lanceolata* [20].

NO is a free radical in the cardiovascular, nervous, and immune systems. It maintains intracellular homeostasis, transports neurotransmitters, and regulates anti-inflammatory activity and cytotoxicity [43]. However, when a large amount of NO is produced, it has detrimental effects on the body, including vasodilation, cytotoxicity, and tissue damage [44,45]. PGE_2_, like NO, is an important inflammatory mediator primarily involved in inducing pain and fever in damaged areas or tissues and is synthesized by COX-2. Excessive production of PGE_2_ causes excessive immune reactions, sclerosis, Parkinson’s disease, Alzheimer’s disease, and other inflammatory diseases [46]. Tumor necrosis factor-α (TNF-α) is produced by activated macrophages and is known as a host defense factor and inflammatory mediator that affects tumor cells. IL-6, a cytokine that causes inflammation, acts as an endogenous pyrogen and has a variety of effects on the immune system and hematopoiesis [47]. In our study, phenylheptatriyne (**1**) and 4′,7-dimethoxyflavanone (**3**) showed excellent inhibitory effects on NO production. Further investigation of the phenylheptatriyne (**1**) and 4′,7-dimethoxyflavanone (**3**) revealed similar effects on PGE_2_, TNF-α, and IL-6. However, since the yield of 4′,7-dimethoxyflavanone (**3**) was 0.8 mg, further studies could not be performed (Figure 9). Therefore, only phenylheptatriyne (**1**) was chosen for investigation of additional anti-inflammatory effects.

The expression of iNOS is induced at the stage of gene transcription by various cytokines released by intracellular bacterial toxins or inflammatory and immune responses, ischemia, tissue damage, oxidative stress, etc., resulting in large amounts of NO [48]. iNOS expression seriously impairs the pathophysiology of the disease. COX-2 is involved in the inflammatory response, blood clotting, vascular regulation, and immune responses, and is activated when inflammatory cytokines are released [49]. In addition, COX-2 induces the production of large amounts of prostaglandins in inflammatory and malignant tumor tissues compared to normal cells, promoting blood vessel formation and cell proliferation, and suppressing immunity, providing an environment that does not interfere with the growth of cancer cells [50]. It is also known that the expression of COX-2 is directly related to the pathogenicity of several diseases. We therefore investigated whether phenylheptatriyne (**1**) inhibits the expression of iNOS and COX-2, which produce NO and PGE2. As a result, phenylheptatriyne (**1**) inhibited the expression of the inflammatory proteins iNOS and COX-2 (Figure 5).

COX-2 and iNOS expression is regulated by the transcription factor NF-κB, and under unstimulated conditions, NF-κB is usually separated from the cytoplasm by the inhibitory factor IκB-α. However, under stimulating conditions, NF-κB translocates into the nucleus, activating the expression of inflammatory cytokines and mediators [51,52]. Thus, we investigated whether phenylheptatriyne (**1**) inhibits intranuclear translocation of IκB-α and p65 in LPS-stimulated BV2 and RAW264.7 cells. Phenylheptatriyne (**1**) inhibited the translocation of NF-κB in a concentration-dependent manner (Figure 6).

HO-1 is known as a representative antioxidant enzyme which plays an important role in protecting cells from oxidative stress and is regulated by the transcriptional activity of nuclear factor erythroid 2-related factor 2 (Nrf2) [53,54]. Therefore, we investigated whether phenylheptatriyne (**1**) expresses the antioxidant enzyme HO-1, and whether the expression of HO-1 was increased (Figure 7). Phenylheptatriyne (**1**) was found to induce the expression of HO-1, and it was necessary to investigate whether the expression of HO-1 exhibits anti-inflammatory and anti-neuroinflammatory effects. Therefore, we investigated whether phenylheptatriyne (**1**) exhibited anti-inflammatory and anti-neuroinflammatory effects through HO-1. As a result, it was confirmed that only the phenylheptatriyne (**1**) treatment group inhibited nitrite compared to the phenylheptatriyne (**1**) treatment group treated with the HO-1 inhibitor SnPP in combination (Figure 8). This is the first report on the anti-inflammatory and anti-neuroinflammatory effect of phenylheptatriyne (**1**), isolated from *C. lanceolata* flowers, through HO-1 expression.

## 4. Materials and Methods

### 4.1. Plant Materials

The flowers of *C. lanceolata* (CL19-001) were collected in August 2019 in Bulgap-myeon, Yeonggwang-si, Jeollanam-do. A voucher specimen (CL19-001) was deposited at the Natural Products Chemistry Laboratory, Chosun University (Gwangju, Korea).

### 4.2. Materials and Instruments Used for Isolation

Resins used for open column chromatography (C. C.) were silica gel 60 (40–63 and 63–200 μm, Merck, Darmstadt, Germany), LiChroprep RP-18 (40–63 μm, Merck), Sephadex LH-20 (25–100 μm, Sigma-Aldrich, Saint Louis, MO, USA) and ODS-A (12 nm, S-75 μm, YMC, Kyoto, Japan) was used, and sand (50–70 mesh, Sigma-Aldrich) was used to place the sample. Nuclear magnetic resonance (NMR) spectra (1 and 2-dimensional) 400 MHz (400 MHz for ^1^H, 100 MHz for ^13^C) were obtained on a JNM ECP-400 spectrometer (JEOL, Tokyo, Japan). Correlation spectroscopy (COSY), distortionless enhancement by polarization transfer (DEPT), heteronuclear single quantum correlation (HSQC), and heteronuclear multiple bond correlation (HMBC) were recorded using standard JEOL pulse sequences. 600 MHz NMR spectra were recorded at KBSI-Gwangju Center on a VNMRS 600 MHz NMR spectrometer (Varian, Palo Alto, CA, USA) and chemical shifts are given in ppm (δ). The solvents used in the analysis, CDCl_3_ and DMSO-d_6_ were purchased from Sigma-Aldrich.

### 4.3. Isolation Procedure of Compounds (1–5) from C. lanceolata Flowers

Dried *C. lanceolata* (400 g) was extracted twice with 3 L of 70% ethanol at 80 °C to obtain 40 g of extract. The extract was sequentially fractionated using CH_2_Cl_2_, EtOAc, *n*-BuOH, and H_2_O to obtain 4.482 g of the CH_2_Cl_2_ fraction, 3.663 g of the EtOAc fraction, 8.051 g of the *n*-BuOH fraction, and 22.273 g of H_2_O fraction.

The CH_2_Cl_2_ fraction (4445 mg) was subjected to silica gel column chromatography (C.C.), and elution was performed using a hexane–acetone gradient system (1:0–0:1, *v*/*v*) to obtain seven subfractions (DM1-DM7). Fraction DM-1 was further chromatographed on silica gel C.C. by using a CHCl_3_-acetone isocratic system (10:1, *v*/*v*) to obtain four subfractions (DM11-DM14). Fraction DM11 was further chromatographed on silica gel C.C. by using a CHCl_3_-acetone isocratic system (10:1, *v*/*v*) to obtain compound **1** (DM113, 54.5 mg). Subsequently, the fraction DM-2 was further chromatographed on a silica gel C.C. by using a hexane-acetone gradient system (1:0–0:1, *v*/*v*) to obtain seven subfractions (DM21-DM27). Fraction DM24 was further chromatographed on a silica gel C.C. by using a hexane-acetone isocratic system (2:1, *v*/*v*) to obtain seven subfractions (DM241-DM247). Fraction DM241 was further chromatographed on a C-18 C.C. by using a MeOH-H_2_O gradient system (1:0–0:1, *v*/*v*) to obtain two subfractions (DM2411-DM2412). Fraction DM2411 was further chromatographed on a C-18 C.C. using a MeOH-H_2_O gradient system (2:1–1:0, *v*/*v*) to obtain seven subfractions (DM24111-DM24117), and compound **2** (DM24117, 0.5 mg) was obtained. Subsequently, fraction DM24116 was obtained by HPLC combined with a Kinetex^®^ 5 µm EVO C18 100 Å LC column using a MeOH-H_2_O isocratic system (1:1, *v*/*v*) to obtain compound **3** (DM241161, 0.8 mg) (Figure 9).

The EtOAc fraction (1500 mg) was subjected to silica gel C.C. by elution with a CHCl_3_-MeOH-H_2_O gradient system (10:1:0.1–1:1:0.1, *v*/*v*/*v*) to obtain two subfractions (EA1 and EA2). Fraction EA-1 was further chromatographed on ODS-A C.C. using a MeOH-H_2_O gradient system (1:3–1:1, *v*/*v*) to obtain six subfractions (EA11-EA16), including compound **4** (EA12, 15 mg). Subsequently, fraction EA14 was further chromatographed on ODS-A C.C. using a MeOH-H_2_O isocratic system (2:3, *v*/*v*) to yield nine subfractions (EA141-EA149). Fraction EA142 was further chromatographed on LH-20 C.C. using a CHCl_3_-MeOH isocratic system (7:1, *v*/*v*) to obtain four subfractions (EA1421-EA1424). Fraction EA1422 was further chromatographed at C-18 C.C. using a MeOH-H_2_O isocratic system (1:1, *v*/*v*) to obtain compound **5** (EA14223, 20.3 mg) (Figure 10).

Compound **1** was obtained as a yellow gum. Based on the analysis of ^1^H- and ^13^C- NMR data, the molecular formula was determined to be C_13_H_8_. ^1^H-NMR (CDCl_3_, 400 MHz): δ: 7.51-7.49 (2H, m, H-2′ and 6′), 7.39-7.29 (3H, m, H-3′, 4′ and 5′), 2.0 (3H, s, H-1); ^13^C-NMR (CDCl_3_, 100 MHz): δ: 4.7 (C-1), 75.3 (C-2), 65.0 (C-3), 67.5 (C-4), 59.0 (C-5), 74.7 (C-6), 78.3 (C-7), 133.0 (C-2′), 128.5 (C-3′), 129.5 (C-4′), 128.5 (C-5′), 133.0 (C-6′).

Compound **2** was obtained as a green gum. Based on the analysis of ^1^H- and ^13^C-NMR data, the molecular formula was determined to be C_18_H_18_O_5_. ^1^H-NMR (CDCl_3_, 600 MHz): δ: 13.54 (1H, s, 2′-OH), 7.86 (1H, d, *J* = 8.4 Hz, H-7), 7.84 (1H, d, *J* = 1.8 Hz, H-6′), 7.44 (1H, d, *J* = 15.6 Hz, H-8) 7.26 (1H, d, *J* = 1.8 Hz, H-2), 7.17 (1H, d, *J* = 1.8 Hz, H-6), 6.50 (1H, d, *J* = 2.4 Hz, H-3′), 6.49 (1H, dd, *J* = 7.2 Hz, H-5′), 3.97 (3H, s, 5-OCH3), 3.94 (3H, s, 4-OCH3), 3.87 (3H, s, 4′-OCH3); ^13^C-NMR (CDCl_3_, 150 MHz): δ: 127.8 (C-1), 151.6 (C-4), 149.3 (C-5), 144.6 (C-7), 123.3 (C-8), 191.7 (C-9), 166.1 (C-1′), 168.6 (C-2′), 101.1 (C-3′), 107.6 (C-4′), 114.1 (C-5′), 110.1 (C-6′).

Compound **3** was obtained as a yellow gum. Based on the analysis of ^1^H- and ^13^C- NMR data, the molecular formula was determined to be C_17_H_16_O_4_. ^1^H-NMR (CDCl_3_, 600 MHz): δ: 7.87 (1H, d, *J* = 8.8 Hz, H-5), 7.41 (2H, d, *J* = 8.4 Hz, H-2′ and 6′), 6.96 (2H, d, *J* = 8.4 Hz, H-3′ and 5′), 6.62 (1H, dd, *J* = 8.8, 2.4 Hz, H-6) 6.48 (1H, d, *J* = 2.4 Hz, H-8), 5.42 (1H, dd, *J* = 16.2, 3.0 Hz, H-2), 3.84 (3H, s, 7-OCH_3_), 3.83 (3H, s, 4′-OCH3), 3.06 (1H, dd, *J*= 16.8, 13.3 Hz, H-3a), 2.80 (1H, dd, *J* = 16.8, 2.9 Hz, H-3b); ^13^C-NMR (CDCl_3_, 150 MHz): δ: 79.8 (C-2), 44.1 (C-3), 190.9 (C-4), 128.7 (C-5), 110.2 (C-6), 166.1 (C-7), 100.8 (C-8), 163.6 (C-9), 114.7 (C-10), 130.6 (C-1′), 127.7 (C-2′ and C-6′), 114.0 (C-3′ and C-5′), 159.9 (C-4′), 55.6 (7′-OCH3), 55.4 (4′-OCH3).

Compound **4** was obtained as a red gum. Based on the analysis of ^1^H- and ^13^C-NMR data, the molecular formula was determined to be C_16_H_14_O_6_. ^1^H-NMR (DMSO-d_6_, 400 MHz): δ: 7.40 (1H, d, *J* = 9.0 Hz, H-5), 6.92 (1H, d, *J* = 1.2 Hz, H-6′), 6.77 (2H, d, *J* = 3.2 Hz, H-2′ and 3′), 6.57 (1H, d, *J* = 8.8 Hz, H-6) 5.42 (1H, dd, *J* = 14.8, 2.4 Hz, H-2), 3.70 (3H, s, 7-OCH3), 3.05 (1H, dd, *J* = 29.2, 12.4 Hz, H-3a), 2.66 (1H, dd, *J* = 19.6, 2.8 Hz, H-3b); ^13^C-NMR (DMSO-d_6_, 100 MHz): δ: 79.8 (C-2), 43.7 (C-3), 190.8 (C-4), 122.6 (C-5), 110.8 (C-6), 157.2 (C-7), 135.9 (C-8), 155.3 (C-9), 114.9 (C-10), 130.5 (C-1′), 118.3 (C-2′), 115.9 (C-3′), 145.7 (C-4′), 146.2 (C-5′), 114.8 (C-6′), 60.7 (OCH3).

Compound **5** was obtained as a red gum. Based on the analysis of ^1^H- and ^13^C-NMR data, the molecular formula was determined to be C_16_H_12_O_6_. ^1^H-NMR (DMSO-d_6_, 400 MHz): δ: 10.84 (1H, s, OH), 9.73 (1H, s, OH), 9.35 (1H, s, OH), 7.45 (1H, d, *J* = 2.0 Hz, H-2′), 7.34 (1H, d, *J* = 8.4 Hz, H-4), 7.26 (1H, dd, *J* = 10.0, 1.6 Hz, H-6′), 6.86 (1H, d, *J* = 8.4 Hz, H-5′), 6.78 (1H, d, *J* = 8.4 Hz, H-5), 4.03 (3H, s, OCH3); ^13^C-NMR (DMSO-d_6_, 100 MHz): δ: 181.9 (C-3), 158.6 (C-8), 158.3 (C-6), 148.7 (C-2), 146.14 (C-3′), 146.06 (C-4′), 132.8 (C-7), 125.1 (C-6′), 123.8 (C-1′), 119.9 (C-2′), 118.4 (C-5′), 116.6 (C-4), 115.3 (C-9), 113.9 (C-10), 112.8 (C-5), 61.3 (7-OCH3).

### 4.4. Cell Culture and MTT Assay

Murine microglia BV2 cells and RAW264.7 cells were donated by Prof. Youn-Chul Kim, Wonkwang University (Iksan, Korea). Cells (5 × 10^6^ cells/dish) were seeded in 100 mm dishes in α-MEM (BV2) or RPMI-1640 (RAW264.7) containing streptomycin (100 μg/mL), 10% heat-inactivated FBS, and penicillin G (100 units/mL), and then incubated at 37 °C in a humidified atmosphere (5% CO_2_ and 95% air). All compounds were dissolved in dimethyl sulfoxide (DMSO) and used in each experiment. To determine cell viability, cells were maintained at a density of ~2 × 10^4^ cells/well and then treated with compounds. After incubation for the indicated times, the cell culture medium was removed from each well and replaced with 200 μL of fresh medium in each well. The cells were incubated with 0.5 mg/mL of 3-(4,5-dimethylthiazol-2-yl)-2,5-diphenyltetrazolium bromide (MTT) for 1 h, and the formed formazan was dissolved in DMSO. The absorbance of the dissolved formazan was measured at a wavelength of 540 nm by using an ELISA microplate reader (Molecular Devices, San Jose, CA, USA).

### 4.5. Measurement of Nitrite Oxide (NO) Generation

To evaluate the production of nitrite oxide (NO), mouse microglia BV2 cells and mouse macrophage RAW 264.7 cells were treated with lipopolysaccharide (LPS) and the five compounds. After incubation for 18 h, the supernatant was mixed with the Griess Reagentand reacted. The measurements were then performed using an ELISA microplate reader at a wavelength of 570 nm (Molecular Devices).

### 4.6. Prostaglandin (PGE_2_) Assay

The culture medium was collected, and the level of PGE_2_ present in each sample was determined using a commercially available kit from R&D Systems (Minneapolis, MN, USA). The assay was performed according to the manufacturer’s instructions.

### 4.7. Interleukin (IL)-6 and Tumor Necrosis Factor (TNF)-α Assay

The culture medium was collected, and the levels of IL-6 and TNF-α present in each sample were determined using a commercially available kit from BioLegend (San Diego, CA, USA). The assay was performed according to the manufacturer’s instructions.

### 4.8. Western Blot Analysis and the Extraction of Cytoplasmic and Nuclear Cell Fractions

Western blotting was performed as follows. After harvesting, the cells were pelleted by centrifugation for 3 min. Next, the cells were washed with PBS and lysed in 20 mM Tris-HCl buffer (pH 7.4) containing a protease inhibitor mixture (0.1 mM phenylmethanesulfonyl fluoride, 5 mg/mL aprotinin, 5 mg/mL pepstatin A, and 1 mg/mL chymostatin). Protein concentration was determined using a Lowry protein assay kit (Sigma-Aldrich). Protein (30 mg) from each sample was resolved using 7.5% and 12% sodium dodecyl sulfate-polyacrylamide gel electrophoresis (SDS-PAGE), and then electrophoretically transferred onto a Hybond enhanced chemiluminescence (ECL) nitrocellulose membrane (Bio-Rad, Hercules, CA, USA). The membrane was blocked with 5% skimmed milk and sequentially incubated with the primary antibody and a horseradish peroxidase-conjugated secondary antibody, followed by ECL detection (Amersham Pharmacia Biotech, Piscataway, NJ, USA). The following antibodies were used: IκBα, NF-κB p65, β-actin (Cell Signaling Technology, Danvers, MA, USA), HO-1 (Merck), iNOS (Cayman Chemical, Ann Arbor, MI, USA), PCNA (Millipore, Middlesex, MA, USA), and Anti-COX2/cyclooxygenase 2 (Abcam, Cambridge, UK). In addition, the cytosolic and nuclear fractions were extracted using the Cayman Nuclear Extraction Kit (Cayman Chemical), and each fraction was lysed according to the manufacturer’s instructions.

### 4.9. Statistical Analysis

All data were acquired from three independent experiments and expressed as mean ± SD. Statistical analysis was performed using the GraphPad Prism software version 3.03 (GraphPad Software Inc., San Diego, CA, USA). The mean difference was determined by one-way ANOVA, followed by Tukey’s multiple comparison test, and statistical significance was defined as *p* < 0.05.

## 5. Conclusions

In this study, we isolated five compounds from the flowers of *C. lanceolata* L. and investigated their anti-neuroinflammatory and anti-inflammatory effects on BV2 microglia and RAW264.7 macrophages. The isolated compounds were identified as phenylheptatriyne (**1**), 2′-hydroxy-3,4,4′-trimethoxychalcone (**2**), 4′, 7-dimethoxyflavanone (**3**), 8-methoxybutin (**4**), and leptosidin (**5**) through NMR analysis. Of these, phenylheptatriyne (**1**) was the most effective at inhibiting nitrite and PGE_2_ production in BV2 microglia and RAW264.7 macrophages. In addition, it was confirmed that phenylheptatriyne (**1**) has an inhibitory effect on the production of the inflammatory cytokines IL-6 and TNF-α. Phenylheptatriyne (**1**) also inhibited the expression of the pro-inflammatory proteins iNOS and COX-2 and prevented the translocation of *p*-IκBa and p65. It was also revealed that phenylheptatriyne (**1**) exhibits anti-inflammatory effects by inducing the expression of HO-1. Thus, phenylheptatriyne (**1**) has been proven to be beneficial as an anti-neuroinflammatory and anti-inflammatory agent.

## Data Availability

The data presented in this study are available within the article. Other data that support the findings of this study are available upon request from the corresponding author.
